# The TransFORmation of IndiGEnous PrimAry HEAlthcare Delivery (FORGE AHEAD): economic analysis

**DOI:** 10.1186/s12961-024-01135-5

**Published:** 2024-05-13

**Authors:** Aleksandra Stanimirovic, Troy Francis, Susan Webster-Bogaert, Stewart Harris, Valeria Rac, the LIONESS study group

**Affiliations:** 1 Program for Health System and Technology Evaluation, Toronto General Hospital Research Institute, University Health Network, 10th Floor, Eaton North, 200 Elizabeth Street, Toronto, ON M5G 2C4 Canada; 2 Ted Rogers Centre for Heart Research at Peter Munk Cardiac Centre, Toronto General Hospital Research Institute, University Health Network, Toronto, ON Canada; 3 Institute of Health Policy, Management and Evaluation, University of Toronto, Toronto, ON Canada; 4 Diabetes Action Canada, CIHR SPOR Network, Toronto, ON Canada; 5 Toronto Health Economics and Technology Assessment Collaborative, Toronto General Hospital Research Institute, University Health Network, Toronto, ON Canada; 6 Centre for Studies in Family Medicine, Schulich School of Medicine and Dentistry, Western University, London, ON Canada

**Keywords:** Indigenous populations, Diabetes and its complications, Community care, Economic analysis

## Abstract

**Background:**

Indigenous populations have increased risk of developing diabetes and experience poorer treatment outcomes than the general population. The FORGE AHEAD program partnered with First Nations communities across Canada to improve access to resources by developing community-driven primary healthcare models.

**Methods:**

This was an economic assessment of FORGE AHEAD using a payer perspective. Costs of diabetes management and complications during the 18-month intervention were compared to the costs prior to intervention implementation. Cost-effectiveness of the program assessed incremental differences in cost and number of resources utilization events (pre and post). Primary outcome was all-cause hospitalizations. Secondary outcomes were specialist visits, clinic visits and community resource use. Data were obtained from a diabetes registry and published literature. Costs are expressed in 2023 Can$.

**Results:**

Study population was ~ 60.5 years old; 57.2% female; median duration of diabetes of 8 years; 87.5% residing in non-isolated communities; 75% residing in communities < 5000 members. Total cost of implementation was $1,221,413.60 and cost/person $27.89. There was increase in the number and cost of hospitalizations visits from 8/$68,765.85 (pre period) to 243/$2,735,612.37. Specialist visits, clinic visits and community resource use followed this trend.

**Conclusion:**

Considering the low cost of intervention and increased care access, FORGE AHEAD represents a successful community-driven partnership resulting in improved access to resources.

## Background

Chronic diseases, such as diabetes, cancer, cardiovascular disease and chronic respiratory diseases, are major contributors to a reduced quality of life, increased risk of hospitalization and premature death [1]. These major chronic diseases, along with mood and anxiety disorders, account for about one-third of direct healthcare expenditures in Canada [1, 2].

The chronic disease burden is heightened in Indigenous populations, including First Nations communities, who experience significantly worse health outcomes, with diabetes prevalence rates that are 3–5 times higher than the general population [3–5]. In addition, the rates of complications from diabetes are also 2 to 5 times higher in First Nations communities than the general population [6, 7]. Chronic diseases have also been shown to impact the life trajectory and life expectancy of First Nations population. A Canadian study found that the burden of chronic diseases among 19 First Nations communities was higher at a relatively younger age in comparison to the general population [6, 7]. In some cases, Indigenous populations have been shown to have a life expectancy 10–15 years less than non-indigenous populations [8].

Distrust between Indigenous populations in Canada and the healthcare system remains a significant driver of health inequities [9]. Significant gaps in care and higher rates of adverse health outcomes have also been identified within Indigenous populations due to a myriad of factors, including structural determinants of health, negative stereotyping and stigmatization [10]. A history of colonization that has undermined Indigenous culture and spiritual practices has created a lack of trust within Indigenous populations towards the healthcare system and has given rise to a structural barrier in utilizing the health system [6].

Given the dramatic rise of chronic diseases and gaps in care in Indigenous populations in Canada, a shift from the dominant episodic and responsive healthcare model most common in First Nations communities to one that places emphasis on proactive culturally appropriate prevention and chronic disease management is urgently needed [11].

The progressive nature of these diseases has substantial health and cost implications due to lost productivity and hospitalizations and poses a significant burden on patients, their families, society, and the healthcare system [2, 5]. There is a growing need to modify healthcare systems to improve the implementation, appropriateness, and effectiveness of healthcare delivery [12, 13].

### The TransFORmation of IndiGEnous PrimAry HEAlthcare Delivery (FORGE AHEAD) research program

The TransFORmation of IndiGEnous PrimAry HEAlthcare Delivery (FORGE AHEAD) Research Program, co-designed with First Nations communities across Canada, aimed to improve chronic disease care and access to available resources by developing and evaluating community-driven, culturally-relevant primary healthcare models using quality improvement theory and processes [11]. FORGE AHEAD, initiated in 2013 and described in detail elsewhere, [11] was directed by a strong multidisciplinary and cross-jurisdictional research team that included First Nations community representatives, Indigenous and non-Indigenous healthcare providers, clinician scientists, academic researchers, policy decision-makers, knowledge-users, and collaborators. Many were nationally recognized leaders in their fields and all stakeholders had experience in working collaboratively with First Nations communities. FORGE AHEAD was guided by the principles of OCAP^®^ [OCAP^®^ is a registered trademark of the First Nations Information Governance Centre (FNIGC)] [14] and community-based participatory research (CBPR) [15]. OCAP^®^, standing for ownership, control, access, and possession, asserts that First Nations alone have control over data collection processes in their communities, and that they own and control how this information can be stored, interpreted, used or shared. CBPR ensures culturally appropriate processes by involving communities as equal partners in all phases of the research process from planning through to knowledge exchange. The Model for Improvement [16] was the process used to guide quality improvement (QI). The Model for Improvement is a modest yet powerful method for accelerating improvements by identifying priorities and testing different ideas for change through Plan-Do-Study-Act (PDSA) cycles. The Diabetes Registry and Surveillance System [7, 17] was used to facilitate the development of community diabetes registries and clinical data.

The FORGE AHEAD objectives [11] were achieved through a series of 10 activities, two of which were implementing community and clinical quality improvement initiatives with evaluation including costing.

Including an economic assessment into the evaluation of quality improvement initiatives, such as FORGE AHEAD, is necessary when we seek to determine the sustainability of these programs and to lobby for the potential scaling up of culturally relevant healthcare models to improve trust in healthcare systems and chronic disease management within underserved First Nations communities. Additionally, the costs and cost-effectiveness of this program and others like it have not yet been reported.

### Objectives of this economic assessment

The overarching goal of this assessment is to perform an economic evaluation to determine the cost of the FORGE AHEAD QI intervention and its implementation, and whether the QI intervention was cost-effective compared with standard diabetes care using a pre-post design and measured by the cost/resource utilization avoided. More specifically, the objectives of this study are to:

1. Identify the relevant resource categories (e.g., costs associated with organizing and operating the initiative); (descriptive analysis of relevant resource categories)
2. Assess resource use including hospital care, specialists’ visits and community care; (descriptive costing analysis)
3. Compare resource utilization and costs with the standard diabetes care provided in the same communities in the pre-intervention period (cost-effectiveness analysis).

## Methods

### Health economic analysis plan

This analysis is a study-based pre-post economic evaluation with two components: a descriptive cost analysis; and a cost-effectiveness study looking at resource utilization and attached costs. The analysis was conducted in accordance with the Consolidated Health Economic Evaluation Reporting Standards (CHEERS) [18] statement while following Canadian Agency for Drugs and Technologies in Health (CADTH) guidelines [19] for economic evaluation in Canada.

### Setting and location

The intervention was implemented in First Nations communities across Canada. The communities were located in Ontario, British Columbia, Alberta, Manitoba, Quebec and Newfoundland and Labrador [11].

Communities were recruited into the program through self-expressed interest in response to personal communication or regional distribution of program information with participation confirmed by a signed research and financial agreement. FORGE AHEAD partnered with 11 communities across six provinces and various isolation levels (Table 1). The program was completed by 9 communities, with 8 communities that collected clinical data using the Diabetes Registry and Surveillance System [7, 17].

**Table 1:** Baseline characteristics of individuals and communities participating in FORGE AHEAD and in the Diabetes Registry and Surveillance System with baseline and follow-up visits [22]

Patient Characteristics (Total *N* = 2008)	
Mean age, years (SD)	60.5 (14.6)
Females: Males, % (*N*)	57.2 (1148): 42.8 (860)
Median duration of diabetes^a^, years (IQR)	8 (3–13)
HbA1c on target (≤ 7.0%)^b^, % (*N*)	30.4 (650)
Blood pressure on target (≤ 130/80)^c^, % (*N*)	20.4 (410)
LDL on target (≤ 2 mmol/L)^d^, % (*N*)	26.5 (532)
Macrovascular complications^e^, % (*N*)	19.9 (400)
Microvascular complications^f^, % (*N*)	17.9 (359)

Community characteristics (Total *N* = 8)	
Isolation level^g^	
Non-isolated, % (*N*)	87.5 (7)
Semi-isolated, % (*N*)	12.5 (1)
Isolated, % (*N*)	0 (0)
Total number of community members	
500–999, % (*N*)	25.0 (2)
1000–4999, % (*N*)	50.0 (4)
5000 and greater, % (*N*)	25.0 (2)
Access to electronic medical records, % (*N*), % (*N*)	62.5 (5)
Onsite primary care physician, % (*N*)	37.5 (3)

Monetary values collected during the study period were converted to 2023 Canadian dollars equivalent

^a^Year of diabetes diagnosis not available for *N* = 429

^b^Baseline HbA1c not available for *N* = 622

^c^Baseline blood pressure not available for *N* = 749

^d^Baseline LDL not available for *N* = 622

^e^Cardiovascular disease, peripheral arterial disease and cerebrovascular disease

^f^Retinopathy, nephropathy and neuropathy

^g^As identified by the Government of Canada

### Study population

The study population consisted of all adults (≥ 18 years) diagnosed with type 2 diabetes (T2DM) from FORGE AHEAD community partners who were registered and had clinical data in the Diabetes Registry and Surveillance System.

Specific settings and locations were considered as FORGE AHEAD team partnered with 11 First Nations communities across six provinces (British Columbia, Alberta, Manitoba, Ontario, Quebec, Newfoundland and Labrador) and three isolation levels (isolated, non-isolated, and remote-isolated/semi-isolated).

### Intervention description

The FORGE AHEAD quality improvement program was implemented in two 18-month waves: 5 communities in wave 1 and 6 communities in wave 2 [11]. Each community assembled both a clinical team and a community team and each team was led by a FORGE AHEAD-trained Community Facilitator. The teams completed readiness questionnaires and attended a series of three quality improvement workshops. The workshops provided quality improvement training and fostered a culture of change via plenaries with expert presenters, teams sharing community strengths and challenges, and breakout sessions where teams planned QI initiatives. The workshops were interspersed with 3-month action periods when the teams implemented their quality improvement initiatives with coaching support. FORGE AHEAD-trained Community Data Keepers managed the Diabetes Registry and Surveillance System, collecting baseline and follow-up patient-level clinical information and entered data on specialist visits, hospitalizations and the use of other community resources [11].

### Study perspective and comparators

The study was undertaken using a payer perspective, using the Ontario Ministry of Health (OMH) as the payer. The comparator was the diabetes care received prior to the intervention implementation [20].

### Time horizon and discount rate

We considered the lifetime of the QI intervention period (18 months). In line with CADTH guidelines [19], we did not consider a 1.5% discount rate as there were no costs and consequences that went beyond 1 year.

### Selection, measurement and valuation of outcomes

Outcomes for the descriptive cost analysis were the total cost of intervention’s implementation and the cost per patient. In the second part of the economic evaluation, pre-post downstream resource utilization, the primary outcome was all-cause hospitalizations (with Length of Stay). Secondary outcomes were: all-cause specialist visits; clinic visits; and community resource use. Clinic visits included: interactions with Community Health Representatives; visits with a Nurse Practitioner; physician visits; visits with a Registered Dietitian; and visits with a Registered Nurse. Community resource use included: counselling for addiction; dialysis treatment; foot care procedures; mental health programs; nutrition programs; physical activity and healthy weight initiatives; psychosocial counselling; smoking cessation programs; substance abuse awareness programs; and wound care clinics.

### Measurement and valuation of resources and costs

Data source included study-derived data and relevant published literature. For the descriptive cost analysis, costing data came directly from the FORGE AHEAD program, which provided detailed information on all costs. In the second part of the economic evaluation—pre-post downstream resource utilization—Data sources were derived from relevant published sources. More specifically, costs related to hospitalizations were estimated using Canadian Institute of Health Information (CIHI) resources. Costs for specialist visits were obtained using the 2021 Ontario Health Insurance Plan (OHIP) billing data. While FORGE AHEAD was implemented across Canada, in this assessment we took on the perspective of the OMH and therefore used OHIP billing data. Please note that as podiatrist services are not covered by OHIP, we used the average cost (Can$90.00) of a podiatrist’s visit as covered by private insurance in Ontario.

### Data collection

For the descriptive cost analysis, we collected the costs in broad categories: staff (e.g., salaries, fee for services); equipment (e.g., equipment and depreciation costs, software costs, installation costs, and maintenance costs); communication (e.g., data transmission costs, modem costs, networking costs); administration (e.g., administration costs, supplies); and healthcare resource utilization.

For the pre-post downstream resource utilization analysis, we used a bottom-up approach to tally healthcare resource utilization costs over the study duration. Healthcare resource utilization costs consisted of hospitalizations and other relevant services as decided with the study stakeholders.

### Currency, price date, and conversion

All costs are reported in 2023 Canadian dollars (2023Can$) using the Bank of Canada Consumer Price Index for inflation to a given base year [21].

### Analytical methods

A study based economic evaluation has two parts. The first is a descriptive cost analysis of the aggregate and direct medical costs associated with the FORGE AHEAD QI initiative (including all QI activities). The second is a cost-effectiveness analysis to estimate whether the adoption of the FORGE AHEAD program represents a beneficial use of OMH resources. We assessed the cost-effectiveness of FORGE AHEAD as incremental differences in cost and number of resources utilization events (before and after program implementation). We did not consider a traditional cost-effectiveness approach where we only perceive lower costs and increased effectiveness in a positive sense.

In this study, we considered the intervention effective if it improved access and quality of patient care among participating communities, reflecting that increased use of resources results from improved equity and increased access to care resources in First Nations communities. The pre-intervention period was defined as May 2015 to April 2016 and the post-intervention period as May 2016 to May 2017.

## Results

### Patient demographics

In Table 1, we present characteristics of the 8 communities who used the Diabetes Registry and Surveillance System and the individuals (*N* = 2810) entered into the System who had information for both baseline and follow-up visits. Most communities (87.5%) were not isolated, had less than 5000 members (75%), and used electronic medical records (62.5%). Only 37.5% of communities had an onsite primary care physician. Individuals were on average 60.5 years old; 57.2% female; and had a median duration of diabetes of 8 years.

### Descriptive analysis of relevant resource categories

Descriptive costs included program implementation costs incurred during the in-person and video conference workshops in participating First Nations communities. The total cost of program implementation over 18 months was $1,221,413.60. When we took into account the total population residing in the 11 communities (*N* = 43,793) where the FORGE AHEAD program was implemented, the cost per person was $27.89 (Table 2). Labour and staff salaries were the main driver of cost.

**Table 2:** Implementation costs (2023Can$)

Variables	Values
Costs FORGE AHEAD Program 18-month	$1,221,413.60
Total population	43,793
Cost/person	$27.89
Total population in registry with baseline data	2810
Cost/person	$434.67

Monetary values collected during the study period were converted to 2023 Canadian dollars equivalent

The cost per person rose to $434.67 (Table 2) when the calculation was limited to the 8 communities with 2810 individuals listed in the Diabetes Registry and Surveillance System with baseline data.

### Descriptive analysis of resource use: specialists’ visits vs community care

#### Primary outcome—all-cause hospitalizations (and length of stay)

As noted in Table 3 there were 8 hospital admissions in the pre-intervention period and 243 hospital admissions in the post-intervention period. On average, the cost of hospital admission increased from $8,240.64 in the pre-intervention period to $10,639.26. The average patient length of stay in the hospital was 9.7 days in the pre-intervention period, with an average patient’s length of stay of 6.9 days in the post-intervention period. There was a minimal change to median hospital length of stay (from 4 to 3.5 days respectively) (Table 3).

**Table 3:** Hospital admissions and length of stay

Number and cost of Hospital admission		
	Pre-intervention (N,$)	Post-intervention (N,$)
Diabetes	0	73
Non-diabetes	8	170
Total Can$2023	8 (cost $68,765.85)	243 (cost $2,735,612.37)
Mean cost in Can$2023	$8240.64	$10,639.26
Range	($5589.25–$8100.00)	($1005.42–$46,642.00)
Length of stay hospital admission (days)		
Mean	9.7	6.9
Median	4.0	3.5
St Dev	13.83	9.66
Range	(1–51)	(1–99)

Monetary values collected during the study period were converted to 2023 Canadian dollars equivalent

In Table 4, we provide information on the top reasons for hospital admissions. The majority of hospitalizations in both pre- and post-periods were non-diabetes related. We note that diabetes-related complications (*N* = 44), including diabetic ketoacidosis foot ulcers, cellulitis, gangrene, foot infections, necrotic toes, septic shock secondary to necrotizing fasciitis and osteomyelitis diabetic foot, remained the top reasons for hospital admissions.

**Table 4:** Top reasons for hospital admissions

Reason of hospital admission	Number (N)
***Diabetes-related complications*** including diabetic ketoacidosis; foot ulcers; cellulitis; gangrene; foot infections; necrotic toes; septic shock secondary to necrotizing fasciitis; osteomyelitis diabetic foot	44
***Abdomina*l** related including abdominal pain; pancreatitis; cholelithiasis; GERD; colonoscopy; incarcerated ventral hernia; hepatic encephalopathy; ulcerative proctitis, viral gastroenteritis; IBD; constipation	35
***Cardiovascular*** including acute coronary syndrome; STEMI; heart failure; cardiac arrest, hypertension	25
***Substance (alcohol) abuse*** related	25
***Respiratory*** related COPD; pneumonia; pulmonary fibrosis; pulmonary embolism; respiratory tract infections; sleep apnea; shortness of breath; bronchopneumonia	25
***Urology*** related urinary tract infections; vaginal atrophy; obstructive prostate; urosepsis	15
***Diabetes*** including non-compliance diabetes; hypoglycemia; hyperglycemia	13
***Kidne*y** related including renal failure; renal insufficiency; nephrotic syndrome; renal failure; end stage renal disease; pyelonephritis	12
***Menta****l health* related panic attack; adjustment disorder; depression; failure to cope	8
***Cancer*** related metastatic cancer; ductal carcinoma; chemotherapy	3

Monetary values collected during the study period were converted to 2023 Canadian dollars equivalent

In Table 5, we present the findings related to the cost of hospitalizations, where we note that majority of hospitalizations in this analysis occurred in the post-intervention period. The total cost of the program implementation and hospital admissions in the post-intervention period was $3,957,025.97, or a cost per person of $90.35.

**Table 5:** Cost of intervention implementation and Primary Outcome (all-cause hospitalizations)

Line		Costs (2023Can$)	Notes
1	Program cost to run FORGE AHEAD (18 months at Western University)	$1,221,413.60	
2	Total population	43,793	
3	Cost/person	$27.89	Line 1/Line 2
4	Total number of hospitalizations	251	
5	Total hospitalizations cost	$2,804,378.23	Line 7 + Line 9
6	Total number of hospitalizations (pre-intervention)	8	
7	Total cost of hospitalization (pre-intervention)	$68,765.86	
8	Total number of hospitalizations (post-intervention)	243	
9	Total cost of hospitalization (post-intervention)	$2,735,612.37	
10	Total cost of intervention implementation and Primary Outcome (post-intervention)	$3,957,025.97	Line 1 + Line 9

Monetary values collected during the study period were converted to 2023 Canadian dollars equivalent

#### Secondary outcomes—specialist visits, clinic visits and community resource use

Table 6 displays trends in the use of other care resources: specialist visits; clinic visits; and community resource use. There was an increase in use health resources from the pre-intervention period to post-intervention period. Specialist visits, clinic visits and community resource use rose from 222 (pre) to 301 (post), 1853 (pre) to 2091 (post) and from 358 (pre) to 578 (post) respectively. Related to specialist visits, the main drivers of cost in the pre-intervention period were ophthalmologist visits, cardiologist visits and surgical consults. The main drivers of cost in the post-intervention period were cardiologist visits, endocrinologist visits and ophthalmologist visits. In reference to clinic visits, the main drivers of cost in the pre-intervention period were consultations with Nurse Practitioners and Community Health Representatives. In the post-intervention period, the main drivers of cost were physicians’ visits and consultations with Nurse Practitioners. The main drivers of cost in community resource use in the pre-intervention period included foot and wound care appointments. Foot care and wound care remained the main drivers of cost in the post-intervention period.

**Table 6:** Secondary outcomes –specialist visits, clinic visits and community resource use

	Pre-intervention		Post-intervention	
	*N*	Cost	*N*	Cost
Specialist visits				
Cardiologist	44	$6242.25	86	$9362
Endocrinologist	14	$2157.5	53	$5977.25
Internist	7	$736.75	16	$1684
Nephrologist	2	$268.15	20	$2162.95
Neurologist	13	$934.45	35	$3066.9
Ophthalmologist	90	$10,034.1	49	$4895.95
Podiatrist	–	–	19	$1710
Surgeon	52	$3968.4	23	$1440.6
Total Can$		$24,341.60		$30,300
Total (2023Can$)	222	$27,378.42	301	$34,080.12
Clinic visits				
Community health representative	752	$14,912.16	532	$10,549.28
Nurse practitioner	802	$21,365.28	526	$14,012.64
Physician	24	$1440	457	$27,420
Registered dietician	3	$107.61	143	$5129.41
Registered nurse	272	$3450.32	433	$5492.60
Total Can$		$41,275.37		$62,603.93
Total (2023Can$)	1853	$46,425.28	2,091	$67,443.52
Community resource use				
Counselling for addiction	–	–	15	$1950
Dialysis treatment	1	$59.8	–	–
Foot care	221	$19,890	281	$25,290
Mental health programs	9	$1305	13	$1885
Nutrition program	7	$125.54	86	$1542.41
Physical activity and healthy weights	5	$179.35	21	$753.27
Psychosocial counselling	–	–	4	$580
Smoking cessation program	–	–	2	$66.90
Substance abuse awareness	–	–	2	$130
Wound care	115	$2362.1	154	$3163.36
		$23,921.79		$35,361
Total (2023Can$)	358	$26,906.50	578	$39,712.17

Monetary values collected during the study period were converted to 2023 Canadian dollars equivalent

### Cost-effectiveness analysis (pre-post resource utilization)

As illustrated in Table 5, there was an increase in both the number and cost (N,$) of hospital admission visits from 8/$68,765.86 (pre-intervention period) to 243/$2,735,612.37 (post-intervention period).

There was an increase in use and costs (N,$) of specialist visits; clinic visits; and community resource use from the pre-intervention period to post-intervention period. More specifically, specialist visits, clinic visits and community resource use rose from 222/$27,378.42 (pre) to 301/$34,080.12 (post), 1853/$46,425.28 (pre) to 2091/$70,415.00 (post) and from 358/$26,906.50 (pre) to 578/$39,712.17 (post) respectively (Table 6).

## Discussion

This analysis is the first study to assess the cost of implementation of a community-driven, culturally relevant QI program and its impact on resource utilization in First Nation communities residing across Canada. The total cost of FORGE AHEAD implementation was $1,221,413.60 and cost/person $27.89 where labour and staff salaries were considered the main driver of cost. We note an increase in both the number and cost (N,$) of hospital admission visits from 8/$68,765.85 (pre period) to 243/$2,735,612.37 (post period). Results illustrate that specialist visits, clinic visits and community resource use followed this trend. Notably, there was an increase in resource utilization in the post-intervention period. Post-intervention impact on the increased use of healthcare resources should not be interpreted in the traditional cost-effectiveness context, where we assume decrease in costs and increase in effectiveness in a positive sense. Rather, they should reflect that increased use of resources resulted in improved equity and access to care resources in the participating First Nation communities.

FORGE AHEAD was the first Canadian study to demonstrate that a partnership with local clinical and community teams implementing a QI intervention can lead to improvements in diabetes management. The authors found that individuals were more likely to receive ≥ 75% of clinical practice guideline (CPG) recommended services compared to baseline (OR: 1.51; 95%CI: 1.27, 1.80) [22].

Findings from the FORGE AHEAD study align with other parts of the world that used a QI approach to target chronic disease care in Indigenous settings [23]. The ABCD study (Australia) demonstrated improvements in delivery of diabetes services across several health centres through three annual cycles. The initial ABCD report (2007) summarized results from 12 health centres indicated an improvement in HbA1c testing from 41 to 72% and an increase in the proportion of people at target HbA1c (< 7.0%) from 19 to 28% after two annual cycles. A subsequent ABCD report (2011) indicated that delivery of overall preventative services increased across 36 health centres through three annual cycles of continuous QI from 31 to 44% and delivery of diabetes services increased from 57 to 63% with improvements sustained in the fourth annual cycle. Contrary to ABCD study, in FORGE AHEAD we did not observe significant change in HbA1c. Similar to the ABCD study, the Indian Health Services (IHS) national T2DM audit and feedback program in the United States has demonstrated a decrease in mean HbA1c from 9% in 1996 to 8.1% in 2014 [24], representing yet another example how a community-led intervention can improve diabetes management. The IHS Division of Diabetes, Area Diabetes Consultants and the Tribal Leaders Diabetes Committee work with the Special Diabetes Programs for Indians (SDPI) grants to provide diabetes treatment and prevention services to IHS, Tribal and Urban Indian health programs [25]. Contrary to FORGE AHEAD which has been conceptualized as community-driven national research program that partners with Indigenous communities in Canada to improve chronic disease care and *access* to available resources whereas IHS is designed as a *process to assess care and health outcomes* for American Indians with diagnosed diabetes. Inherently FORGE AHEAD AND IHS may be serving a different purpose, resulting in different outcomes.

The increasing demand on the healthcare system to deliver evidence-based practice with scarce resources has led to a need to evaluate the cost-effectiveness of healthcare improvement and knowledge translation strategies [26]. This economic assessment found that the FORGE AHEAD program improved access to quality care and reduced underutilization of health services among First Nations communities. Underutilization of health services among chronic non-communicable disease sufferers, such as diabetes mellitus (DM), is recognized as a significant contributing factor to increased morbidity and mortality. Underutilization of health services can be perceived as failure to adopt an affordable health service that is highly possible to improve the quality or quantity of life [27]. It manifests behaviorally when individuals do not seek medical care when feeling ill or suspecting they should go [28, 29]. Additionally, the underuse of health services lowers process-related healthcare costs in the long term because the necessary care is not provided, conversely, increasing the use of health services increases short-term costs [29]. Improvements in the quality of care provided will require an increase in costs initially; however, improvements could lower long-term costs by slowing disease progression, which may reduce complications and hospital readmissions [30, 31].

**Fig. 1:**
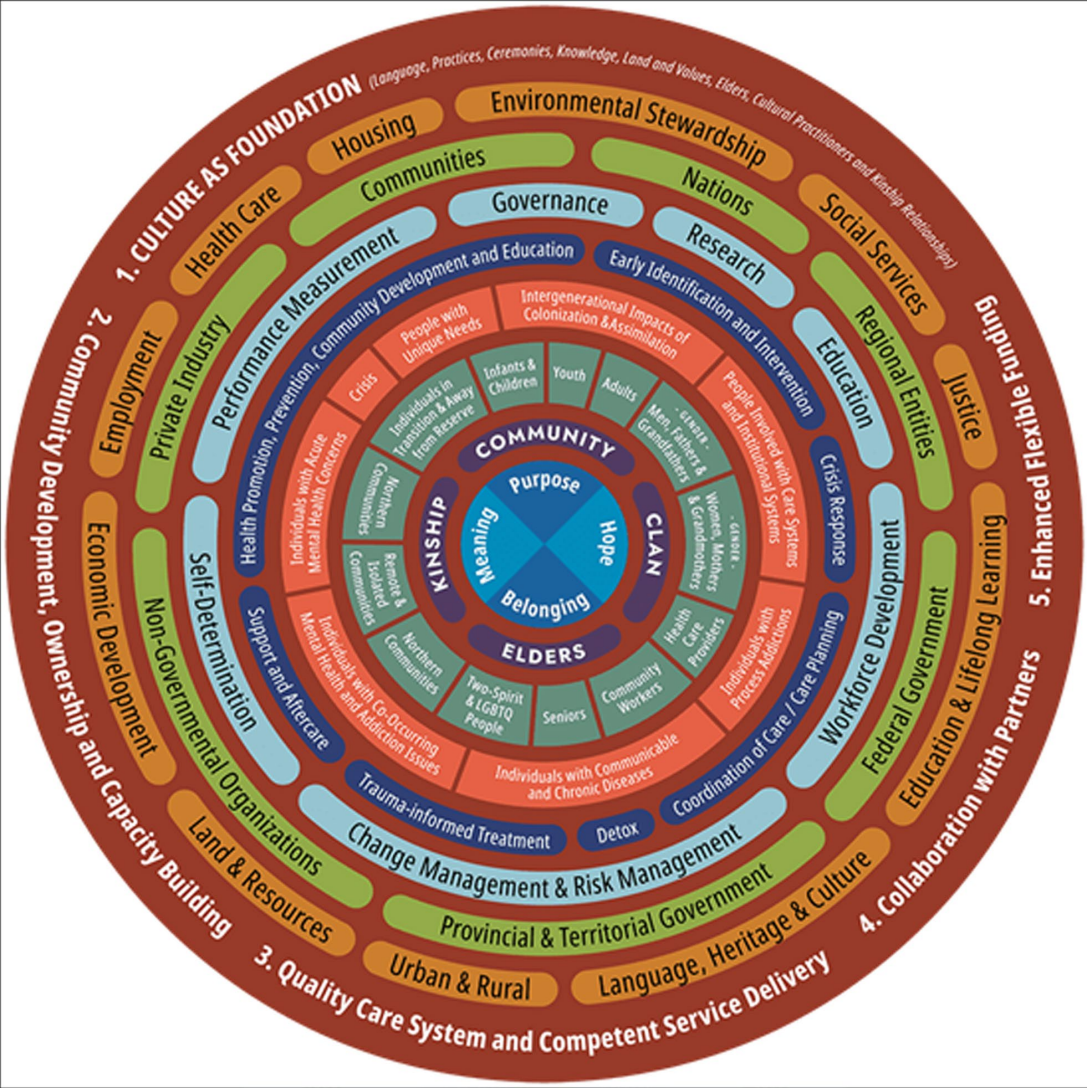
First Nations Mental Wellness Continuum (FNMWC)

Current economic frameworks lack many elements important to the worldview of Indigenous communities. An example of a framework that does include these elements is the First Nations Mental Wellness Continuum (FNMWC) (Fig. 1) [32]. FNMWC is a national framework that addresses mental wellness through culturally safe delivery of services among First Nations communities in Canada. While the framework is focused on mental health, it clearly depicts the tiers, complexities, and intricacies of Indigenous community involvement as interventions and programs are implemented, based on: Culture as a Foundation; Community Development, Ownership and Capacity Building; Quality Care Systems and Competent Service Delivery; Collaboration with Partners and Enhanced Flexible Funding. It supports culturally safe delivery of services. One of the key elements emphasized through FNMWC is the degree/level of Community Involvement to promote change. The economic evaluation of the FORGE AHEAD initiative, similarly, incorporated the community perspectives and how the intervention influences resources utilization at the Community level. FORGE AHEAD was co-designed with leaders from First Nation communities. In Indigenous settings, following the principles of OCAP^®^ is critical to facilitate the participation and development of the community to result in effective and culturally relevant clinical strategies/programs to improve chronic disease outcomes. Yet there remains a gap in how we evaluate Spiritual health using an economic lens. Future work should consider inclusion of cultural sensitivity.

We propose a de novo Decision Determinants (DD) framework, Health Technology Assessment (HTA) framework for the evaluation of programs/interventions implemented in Indigenous populations [33]. The DD framework is similar in several respects to Health Technology Assessment (HTA) frameworks but is based on an explicit set of social values. HTA (evidence based medicine, economics, and bioethics/social science) is used to aggregate decision attributes and is rooted in a theoretical framework of optimal decision making rather than one related to broad social goals, such as health or welfare maximization. The purpose of the DD framework is to embrace a more holistic approach while considering the Spiritual lens of Indigenous populations. The framework will integrate the community aspect in evaluation, resulting in achievement of common goals, promoting interaction among members to address member concerns with sensitivity meanwhile celebrating heritage and tradition. Finally, for successful intervention implementation and adoption in Indigenous populations, it is fundamental to co-design and co-evaluate programs and interventions in partnership with an Indigenous evaluator.

## Conclusion

Indigenous populations living in Canada are among the highest-risk populations for diabetes and related complications. Yet there are significant inequities in access to diabetes healthcare and outcomes for these Indigenous populations. Poor success of many diabetes management strategies highlights the limitations of health services when they are socially, culturally, and contextually irrelevant. Future studies need to include a more holistic approach and community involvement, with the DD framework being culturally sensitive to Indigenous populations by considering the value in combining elements of Western/Indigenous medicine.

FORGE AHEAD is the first Canadian study to demonstrate that a community-led QI intervention can improve diabetes management and healthcare access. Considering the intervention’s low cost and its potential to improve equity and access to care, FORGE AHEAD epitomizes a successful community-driven and culturally based partnership resulting in improved equity and access to resources in participating communities.

## Data Availability

The datasets generated during and/or analyzed during the current study are not publicly available to respect the wishes of our partnering First Nations communities and individual participants.
